# Neurally adjusted ventilatory assist (NAVA) versus pressure support ventilation: patient-ventilator interaction during invasive ventilation delivered by tracheostomy

**DOI:** 10.1186/s13054-018-2288-2

**Published:** 2019-01-07

**Authors:** Olivier Lamouret, Laure Crognier, Fanny Vardon Bounes, Jean-Marie Conil, Caroline Dilasser, Thibaut Raimondi, Stephanie Ruiz, Antoine Rouget, Clément Delmas, Thierry Seguin, Vincent Minville, Bernard Georges

**Affiliations:** 1 Service de Réanimation Polyvalente, CHU Rangueil, 1 Avenue Jean Poulhès, Pôle d’Anesthésie et Réanimation, TSA 50032, 31059 Toulouse Cedex 9, France; 2 Department of Anaesthesiology and Critical Care Unit, University Hospital of Toulouse, 31059 Toulouse Cedex 9, France

**Keywords:** Neurally adjusted ventilatory assist, Pressure support, Tracheostomy, Difficult weaning, Patient-ventilator asynchronies

## Abstract

**Background:**

Prolonged weaning is a major issue in intensive care patients and tracheostomy is one of the last resort options. Optimized patient-ventilator interaction is essential to weaning. The purpose of this study was to compare patient-ventilator synchrony between pressure support ventilation (PSV) and neurally adjusted ventilatory assist (NAVA) in a selected population of tracheostomised patients.

**Methods:**

We performed a prospective, sequential, non-randomized and single-centre study. Two recording periods of 60 min of airway pressure, flow, and electrical activity of the diaphragm during PSV and NAVA were recorded in a random assignment and eight periods of 1 min were analysed for each mode. We searched for macro-asynchronies (ineffective, double, and auto-triggering) and micro-asynchronies (inspiratory trigger delay, premature, and late cycling). The number and type of asynchrony events per minute and asynchrony index (AI) were determined. The two respiratory phases were compared using the non-parametric Wilcoxon test after testing the equality of the two variances (F-Test).

**Results:**

Among the 61 patients analysed, the total AI was lower in NAVA than in PSV mode: 2.1% vs 14% (*p* < 0.0001). This was mainly due to a decrease in the micro-asynchronies index: 0.35% vs 9.8% (*p* < 0.0001). The occurrence of macro-asynchronies was similar in both ventilator modes except for double triggering, which increased in NAVA. The tidal volume (ml/kg) was lower in NAVA than in PSV (5.8 vs 6.2, *p* < 0.001), and the respiratory rate was higher in NAVA than in PSV (28 vs 26, *p* < 0.05).

**Conclusion:**

NAVA appears to be a promising ventilator mode in tracheotomised patients, especially for those requiring prolonged weaning due to the decrease in asynchronies.

## Introduction

Difficult weaning from ventilator support was defined by the consensus conference published in the *European Respiratory Journal* in 2007 [1]. Nearly half of the patients who fail at the first single-breathing trial still require mechanical ventilation at day 7 [2, 3]. Prolonged weaning significantly increases the incidence of ventilator-associated pneumonia (VAP) [4] and mortality [5].

Prolonged weaning could be caused by respiratory or heart failure, intensive care unit (ICU)-acquired weakness [1, 2], or be related to poor patient-ventilator interaction (called patient-ventilator asynchrony). According to research, tracheostomy could reduce the duration of mechanical ventilation [6, 7] and both ICU [6, 8] and hospital mortality [9].

Patient-ventilator asynchronies occur in approximately 25% of mechanically ventilated patients, thus increasing the duration of mechanical ventilation and the length of ICU or hospital stays [10, 11].

Asynchronies can be divided into macro- and micro-asynchronies. Macro-asynchronies are generally comprised of ineffective triggering, some double triggering and, more rarely, auto-triggering [10, 12]. Micro-asynchronies comprise inspiratory trigger delay, premature, and late cycling. They may reflect a delay between a diaphragm signal and a respirator response [13]. An inspiratory trigger delay is too big a delay between the diaphragmatic signal and respiratory insufflation. Late cycling, initially called “prolonged cycle” [10], is too long a respiratory insufflation while the patient is already in expiration time (diaphragmatic relaxation) [14, 15]. These prolonged inspirations favour other asynchronies such as ineffective triggering and are potentially responsible for dynamic hyperinflation that increases both respiratory work and the duration of the ventilator support [16]. We examined a third micro-asynchrony called premature cycling [13], which is defined by a ventilator insufflation stop while the diaphragmatic signal is still in inspiration.

Pressure support ventilation (PSV) is a normal weaning mode. New assisted proportional ventilation modes such as neurally adjusted ventilator assist (NAVA) could help optimise weaning. NAVA mode is based on the detection of a diaphragm electromyographic signal (Eadi) [17] which triggers the ventilator and provides proportional assistance.

The benefits on patient-ventilator synchrony have been well established on intubated patients [13, 18–22].

To our knowledge, no study has compared the occurrence of various asynchronies in NAVA versus PSV on ventilated tracheotomised patients [23].

The objective of our study was to show a reduction of asynchronies in NAVA compared with PSV in mechanically ventilated patients with tracheostomies. The secondary objectives were to compare the different types of asynchronies between NAVA and PSV and other respiratory parameters.

## Material and methods

We conducted a prospective, sequential, non-randomized, single-centre study in the ICU of the University Hospital of Rangueil in Toulouse, France. The study was approved by our institution’s Ethics Research Committee (No. 50–0614). The inclusions started in June 2014 for a period of 2 years and 9 months. All ventilated tracheotomised patients were eligible for inclusion. The exclusion criteria were: patients younger than 18 years old, pregnant women, classical contraindications to Eadi catheter placement (recent oesophageal or gastric surgery, presence of oesophageal varices), a progressive infectious process defined by the association with clinical examination or imaging of an infected area with systemic inflammatory response syndrome (SIRS), haemodynamic failure (mean arterial pressure less than 65 mmHg or catecholaminergic treatment), decisions to withhold life-sustaining treatment, and the presence of a guardianship.

### Protocol after inclusion

Included patients benefitted from the placement of an Eadi catheter 16 Fr/125 cm by nasal insertion (Eadi Catheter, Maquet Critical Care®, Solna, Sweden*).*

As described by Sinderby et al. [17], the electrical activity of the diaphragm is obtained through an array of electrodes placed in the oesophagus at the diaphragm level. The signal is amplified and acquired into an online processing unit. The processed signal is amplified and output to a Servo-i® ventilator, which delivers assistance in proportion to the diaphragm’s electrical activity.

Positioning of the Eadi catheter was monitored by a special tool implemented in the Servo-i® ventilator.

The correct position of the electrodes in relation to the heart and diaphragm was estimated by assessing the different leads for the presence/absence of the p-wave and QRS complex.

The optimal catheter position was identified through co-existence of three criteria: stable Eadi signal, electrical activity highlighted in the central leads of the catheter’s positioning tool, and absence of a p-wave in the distal lead.

Among the included patients, some were ventilated using NAVA prior to tracheostomy (through an orotracheal tube). For the other patients, the Edi catheter was placed just after the tracheostomy procedure. The baseline mode before inclusion could equally be NAVA or PSV.

After inclusion, the tracheotomised patients were mechanically ventilated according to two 60-min recorded periods in a random assignment: PSV followed by NAVA, or NAVA followed by PSV.

An experienced physician adjusted the optimal ventilation settings tailored for each patient in two phases.

We started with a theoretical phase with major targets such as a low tidal volume (VT), a moderate positive end-expiratory pressure (PEEP), and an expiratory trigger suited to the respiratory disease. This was followed by a dynamic phase observing the curves as detailed below to improve the settings.

For PSV, the level of support was the minimum level to obtain a VT expired (VTe) between 6 and 8 ml/kg of predicted body weight (PBW).

Inspiratory trigger in-flow was adjusted as low as possible without auto-triggering. The expiratory trigger was set between 20% and 50%, depending on the past medical history of a restrictive (low trigger), obstructive syndrome (high trigger), or none (intermediate trigger of 25% to 30%).

A moderate PEEP level seems to be an appropriate compromise during the weaning process.

External PEEP and expiratory cycling were simultaneously optimised through the flow curves of the ventilator, such as the expiratory flow returning to zero which reflects the absence of major auto-PEEP.

The PEEP level was determined for PSV and we used the same level for NAVA.

In NAVA, the “preview NAVA” function of the Servo-i® ventilator was used to estimate the NAVA gain to obtain the same peak pressure as that during PSV, and VT between 6 and 8 ml/kg of PBW. If VTe exceeded 8 ml/kg, we decreased the NAVA level.

The Eadi inspiratory trigger was set to a predetermined default value of 0.5 μV, always above the minimal value of the patient’s Eadi. The cycle-off value was set to 80% of the Eadi peak in the NAVA mode. In NAVA, the first inspiratory trigger detected (pneumatic or neurally) is rewarded by a respirator insufflation.

The Eadi catheter was susceptible to movement. NAVA includes a safety feature. If the Eadi signal exhibits artefacts or is lost, the ventilator automatically reverts to PSV.

We checked the correct position of the Eadi catheter at the start and at the end of the recording. We looked for a stable nose tip measurement and we used the special tool implemented in the Servo-i ventilator to verify the optimal position based on the co-existence of the three criteria, as previously mentioned.

All the settings were kept constant and the same patient body position was used during the entire recording process and for the two modes.

#### Data collection

Data were recorded using Servo-i software CPR (Maquet Critical Care®, Solna, Sweden). Pressure, flow, volume curves, diaphragmatic signals (Eadi), VT, and assistance levels were collected for each patient. If a patient required aspiration, the nurse disconnected the circuit during the recording process which was easy to detect on the records (loss of flow and pressure curve).

#### Data analysis

Two independent physicians analysed the recorded data. In the event of a mismatch, a third physician was used. Analysis of the respiratory curves was performed manually by examining eight recording periods of 1 min at regular intervals of 8 min for each mode, which represented an analysis period of 16 min for each patient.

If one of these 1-min periods showed an artefact (the patient disconnected for any reason, secretions in the ventilator circuit, cough), we analysed the following minute.

**Fig. 1:**
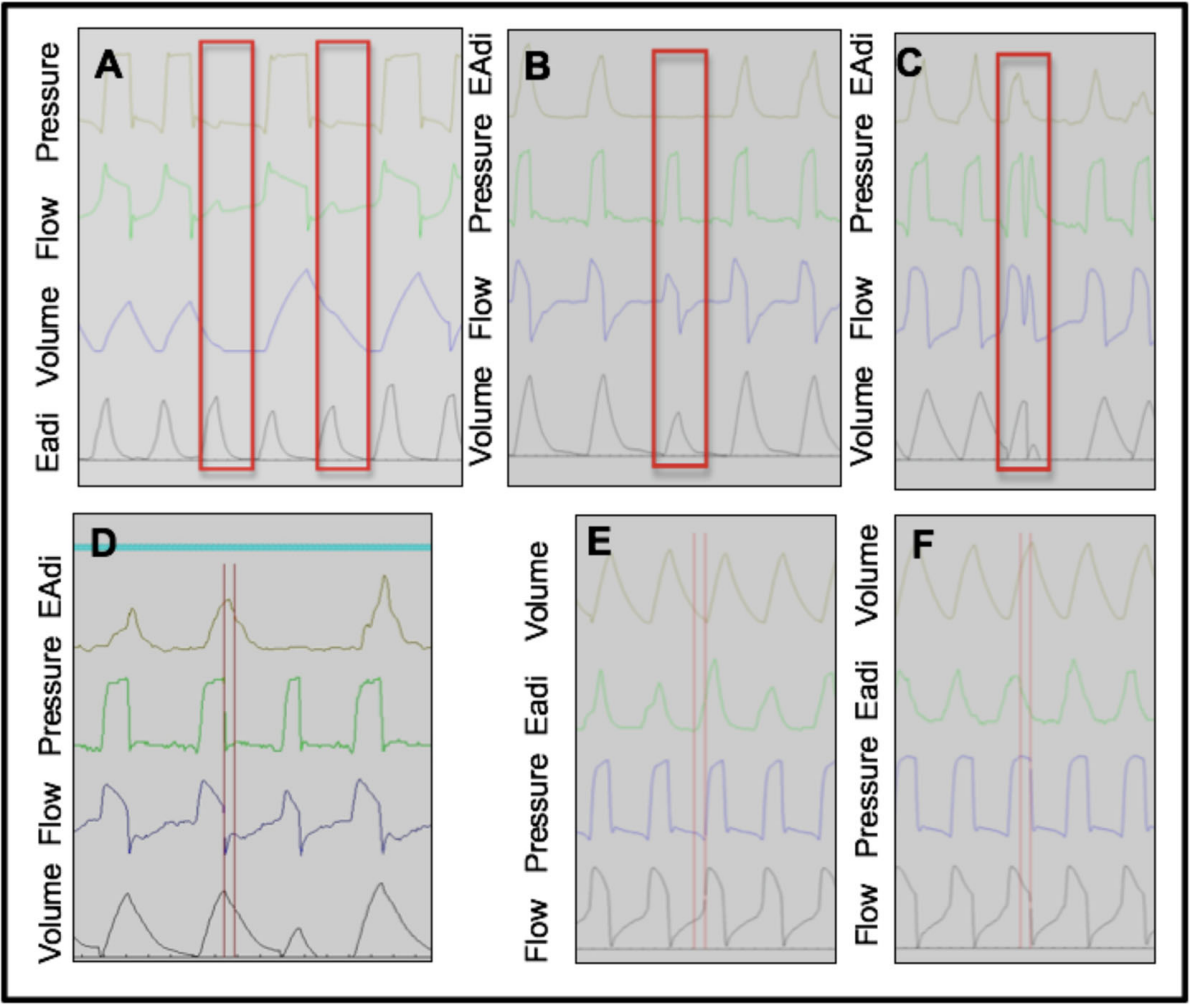
Description of asynchronies studied. **a** Ineffective triggering; **b** auto-triggering; **c** double triggering; **d** premature cycling; **e** inspiratory trigger delay; **f** late cycling. EAdi electrical activity of the diaphragm

Six types of asynchrony were analysed (Fig. 1). Macro-asynchronies have been previously described by Thille et al. [10]: ineffective triggering (Fig. 1a) was defined by the existence of a diaphragmatic signal without a respiratory cycle; auto-triggering (Fig. 1b) was defined by the existence of a ventilator cycle without a diaphragmatic signal; double triggering (Fig. 1c) was defined by the presence of two successive inspiratory cycles without an intermediate expiration or with an interrupted expiration.

Micro-asynchronies were defined by a delay exceeding 200 ms: between diaphragmatic contractions and the start of insufflation—inspiratory trigger (Fig. 1e); between the end of diaphragmatic contractions and the opening of the expiratory valve—late cycling (Fig. 1f); and between the end of diaphragmatic contractions and the opening of the expiratory valve—premature cycling (Fig. 1d).

In the absence of a consensual definition, we used a delay of 150 ms for each type of micro-asynchrony, which represents the conscious perception threshold which could be a source of discomfort [24]. We added a 50-ms safety margin.

The total number of asynchronies was then calculated for each ventilation mode by adding different asynchronies recorded during eight periods of 1 min for a total of 1 h of recording.

An asynchrony index (AI) was calculated. This index corresponds to the total number of asynchrony events divided by the total respiratory rate (which corresponds to the total Eadi signals) × 100 [10, 13]. The AI was expressed as a percentage.

For each subtype of asynchrony, a percentage of asynchronies was calculated as follows: number of asynchrony events divided by the total Eadi signals × 100. In addition, we calculated the VT in ml/kg of PBW, corresponding to the different recording periods. The variability of VT in each ventilation mode was evaluated by the coefficient of variation.

#### Statistical analysis

The characteristics of the population and of the analysed variables are described as medians and 95% confidence intervals. The two respiratory phases were compared using the non-parametric Wilcoxon test after testing the equality of the two variances (F-Test).

The objective was to demonstrate a decrease in the total number of patient-ventilator asynchronies and in AI. Power analysis indicated that a sample size of 28 was sufficient to demonstrate a 20% reduction in the number of asynchronies between the PSV and NAVA modes, with α and β risks of 0.05 and 0.20, respectively.

The analysis was performed with MedCalc®15 statistical software (Ostend, Belgium). The difference between the groups was considered statistically significant for *p* values < 0.05.

## Results

### Population

**Fig. 2:**
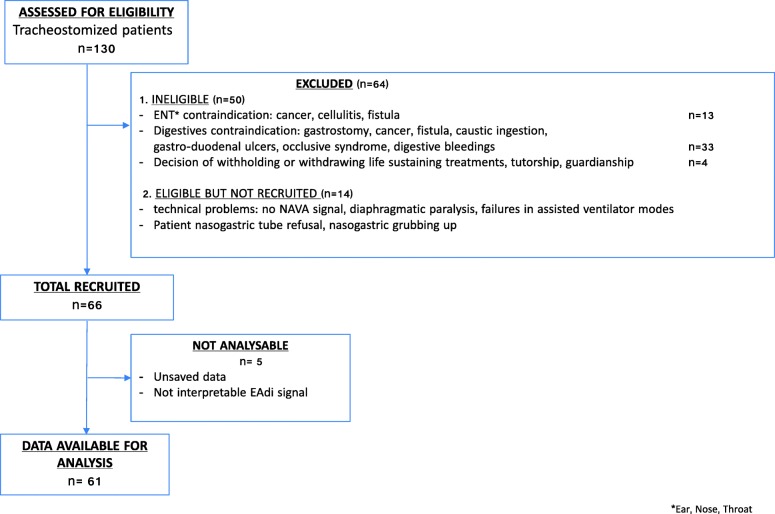
Flow chart of the studied population

A total of 66 out of 130 eligible patients (50.8%) were included (Fig. 2). Three recordings were uninterpretable due to a heavy number of artefacts and two measurements were not recorded. Finally, a total of 61 patients were analysed (46.9%).

The main characteristics of the population are presented in Table 1, where the results are expressed as the median and range and a 95% confidence interval or as a number and percentage.

**Table 1:** Main characteristics of the population

Total population (*n* = 61)	Median	95% CI	Minimum	Maximum
Age (years)	65	63–66.6	32	85
BMI (kg/m^2^)	27.7	25.4–29.5	20.4	39
SAPS II	59	53–69	22	93
Men/women, *n* (%)	44 (72.1%)/17 (27.9%)			
Duration of MV (days)	39	33–44	15	164
Weaning period (days)	21	19–26	2	58
Extubation failure (*n*)	0	0–1	0	4
ICU stay (days)	43	36.4–53	13	169
Respiratory disease, *n* (%)				
COPD	18 (29.5%)			
Restrictive syndrome	6 (9.8%)			
Cardiac disease, *n* (%)				
Coronary disease	19 (31.1%)			
Heart failure	10 (16.4%)			
Obesity, *n* (%)				
Normal weight	21 (34.4%)			
Overweight	19 (31.1%)			
Moderate obesity (BMI 30–35 kg/m^2^)	16 (26.2%)			
Severe obesity (BMI > 35 kg/m^2^)	5 (8.2%)			
Postoperative, *n* (%)				
Digestive surgery	2 (3.3%)			
Cardiothoracic and vascular surgery	22 (36.1%)			
Day 28 mortality, *n* (%)	3 (4.9%)			
Day 90 mortality, *n* (%)	28 (45.9%)			

*BMI* body mass index, *CI* confidence interval, *COPD* chronic obstructive pulmonary disease, *ICU* intensive care unit, *MV* mechanical ventilation, *SAPS II* Simplified Acute Physiology Score II

The population presented with a median Simplified Acute Physiology Score (SAPS) II of 59 (range 22–93). The majority of patients were male (72.1%), with a median age of 65 years (range 32–85). Over one-third of the patients had a known respiratory disease, mainly chronic obstructive pulmonary disease (COPD), and nearly half of the patients had past cardiac medical history. Nearly 46% failed extubation after a positive spontaneous breathing trial (SBT).

The median length of time between ICU admission and tracheostomy was 21 (95% confidence interval 19–26) days with a median time of mechanical ventilation of 39 (95% confidence interval 33–44) days.

All-cause mortality was high; 45.9% of patients (*n* = 28) died during their hospital stay.

### Ventilator settings and asynchronies

Ventilator settings are shown in Table 2. The median level of support was 10 (range 3–24) cmH_2_O in PSV and 0.6 (range 0.1–3) cmH_2_O/μV in NAVA. The inspiratory flow trigger level was set at a median of 6 (range 1—9) (the Maquet trigger range from 1 to 10 corresponds to 3 to 1 l/min). The expiratory trigger was set according to the patient’s respiratory restrictive or obstructive status or by considering a dynamic lung hyperinflation. The median expiratory trigger was set at 30% (range 15–50). PEEP was significantly lower in NAVA than PSV (5 versus 6 cmH_2_O).

**Table 2:** Ventilation settings and asynchronies recorded

Total population (*n* = 61)	PSV				NAVA				*p*
	Median	95% CI	Minimum	Maximum	Median	95% CI	Minimum	Maximum	
Level of support	10^a^	8–12	3	24	0.6^b^	0.5–0.8	0.1	3	NA
Expiratory trigger PSV (%)	30	30–35	15	50	NA	NA	NA	NA	NA
Inspiratory trigger PSV (1–10)	6	5–6	1	9	NA	NA	NA	NA	NA
Macro-asynchronies									
Auto-triggering	0	0–0.2	0	4.1	0	0–0.08	0	24	**0.0182**
% auto-triggering	0	0–0.6	0	18.4	0	0–0.19	0	24	**0.0251**
Double triggering	0.1	0–0.3	0	5.3	0.1	0–0.20	0	24	**0.0223**
% double triggering	0.3	0–0.5	0	16.6	0.4	0–0.79	0	24	**0.0063**
Ineffective triggering	0	0–0.08	0	14.4	0	0–0.13	0	24	0.4363
% ineffective triggering	0	0–0.19	0	33.9	0	0–0.46	0	24	0.3272
Macro-asynchrony index (%)	1.6	1–3.18	0	33.9	1.64	0.4–2.5	0	24	0.6420
Micro-asynchronies									
Premature cycling-off	0.13	0–0.6	0	4	0	0–0	0	24	**< 0.0001**
% premature cycling-off	0.3	0–1.9	0	31	0	0–0	0	24	**< 0.0001**
Late cycling-off	0.123	0–0.38	0	11.4	0	0–0	0	24	**0.0002**
% late cycling-off	0.5	0–1.24	0	27.7	0	0–0	0	66	**0.0013**
Inspiratory trigger delay	0.88	0.5–1.75	0	25.9	0	0–0.13	0	24	**< 0.0001**
% inspiratory trigger delay	3.55	1.8–5.96	0	81.9	0	0–0.4	0	24	**< 0.0001**
Micro-asynchrony index (%)	9.8	6.8–15.7	0	100.7	0.35	0–0.86	0	24	**< 0.0001**
Total asynchronies									
Total AI (%)	14	9.9–21.7	0	101.8	2.1	0.8–3.9	0	24	**< 0.0001**
Patients with AI > 10%		38	62.3%			7	11.5%		
Respiratory rate (cycle/min)	28	25–29	12.6	46	26	25–28	11	46.6	**0.0323**
VT expired (ml)	400	381–418	194.8	947	366	336–388	207.1	982	**0.0020**
Volume/min (l/min)	11.4	10–13	5.5	21.7	11.4	11–12.2	6	20.4	0.8953
VT (ml/kg PBW)	6.2	5.9–6.4	3.1	12.4	5.8	5.3–6.2	3.3	12.9	**0.0009**
PEEP (cmH_2_O)	6	5–6	3	10	5	5–5	3	10	**0.0029**
Eadi peak	15.7	12.4–19.7	3.3	84.75	15.2	12.5–18.2	6.72	84	0.1142

Results are expressed as medians, 95% confidence intervals (CIs) or number (%)

Significant *p* values are shown in bold typeface

*AI* asynchrony index, *Eadi* electrical activity of the diaphragm, *NA* not available, *NAVA* neurally adjusted ventilatory assist, *PDW* predicted body weight, *PEEP* positive end-expiratory pressure, *PSV* pressure support ventilation, *VT* tidal volume

^a^Results expressed in cmH_2_O

^b^Results expressed in cmH_2_O/μV

**Fig. 3:**
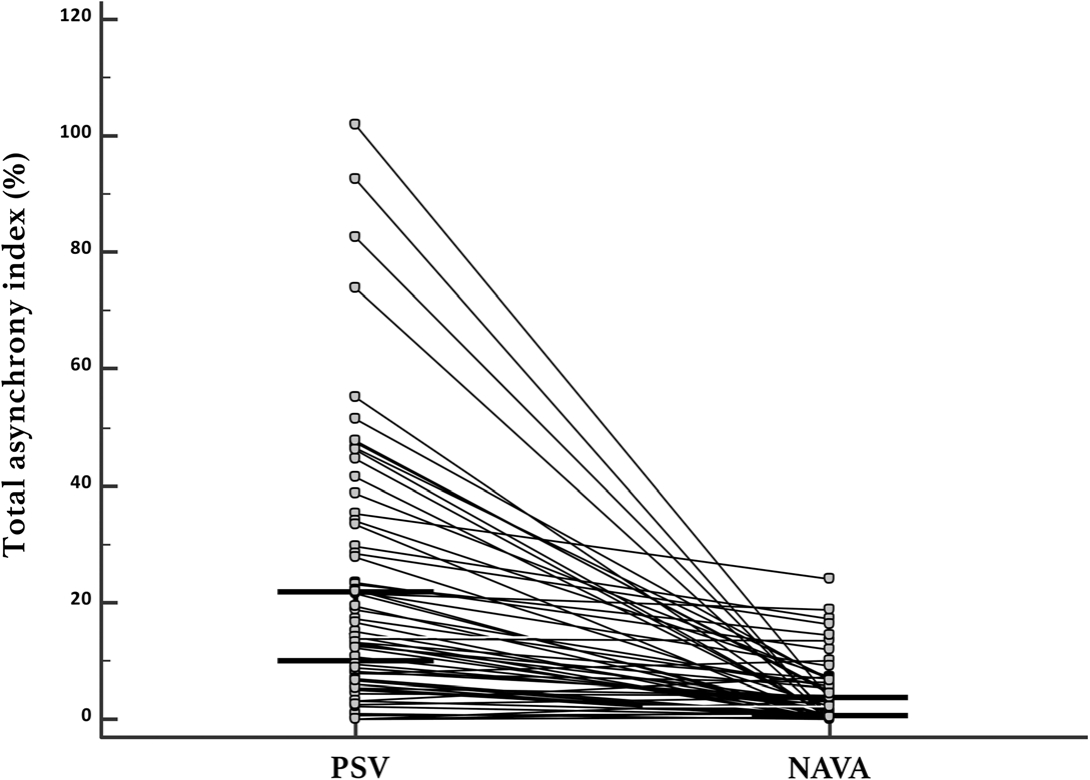
Primary objective; total asynchrony index in neurally adjusted ventilatory assist (NAVA) versus pressure support ventilation (PSV)

The total asynchrony index was lower in NAVA than in PSV (2.1% vs 14%, *p* < 0.0001) (Fig. 3). Similarly, an AI > 10% was less frequent in NAVA than in PSV mode (11.5% vs 62.3%, *p* < 0.0001). Among the macro-asynchronies, there was no difference in auto-triggering and ineffective triggering between the two groups. However, there was more double triggering in NAVA than in PSV (0.4% vs 0.3%, *p* < 0.01).

Regarding micro-asynchronies, there were less of each type in NAVA mode than in PSV mode.

The tidal volume was lower in NAVA than in PSV (5.8 vs 6.2 ml/kg, *p* < 0.001). The respiratory rate was higher in NAVA than in PSV (28 vs 26 breaths/min, *p* < 0.05).

## Discussion

Difficult weaning from ventilator support requires optimal ventilation settings. Here, we demonstrated in a prospective study that NAVA reduced total patient-ventilator asynchronies mainly through a decrease of micro-asynchronies in tracheotomised patients in comparison with the PSV mode.

Generally, tracheostomy is performed in the ICU for difficult weaning and prolonged ventilation or to protect the upper airways in brain-injured patients. In our population, a majority of the patients were tracheotomised for difficult weaning.

Frutos-Vivar et al. [8] showed in a large multicentric observational study that tracheostomy is mainly performed in the context of respiratory diseases. A minority of 22% of the patients included were tracheotomised due to a coma.

In comparison to this previous study, there were several comas (less than 10%), except for post-anoxic coma, because of the coexistence of a neurosurgical ICU in the same University Hospital.

Another specific feature of our study was the important role of patients with cardiac and respiratory diseases, these representing a particular population for whom weaning would be difficult.

We did not precisely quantify the ICU-acquired weakness but, as recently established, weaning failure is not associated with a poor Medical Research Council (MRC) score [25].

Unfortunately, not all the tracheotomised patients were included (almost 50%), with the majority of them presenting contraindications to the nasogastric tube.

We also lost some potentially eligible patients because of the small number of Servo-i® devices in our unit. When a patient benefited from NAVA using another interface (orotracheal tube, non-invasive ventilation (NIV)), it would have been unethical to exchange the machine for the gain of a potentially included patient. When several patients were tracheotomised and several Servo-i® ventilators were available, we favoured patients with the most difficult weaning. In addition, we had to deal with catheter shortages for a few weeks.

Our results exhibited some macro-asynchronies in PSV mode, compared with the 10% reported in the literature [10, 26]. This benefit can be attributed to the combined effects of tracheostomy and a careful personalized ventilator setting.

Thille et al. showed that optimized PSV settings help avoid patient-ventilator asynchronies and are related to a reduced weaning period, even in tracheotomised patients [10]. The same team also demonstrated that reducing pressure support or inspiratory duration eliminated ineffective triggering in a majority of the patients with a high percentage of ineffective efforts [16]. Consequently, we showed no difference in AI between the PSV and NAVA in macro-asynchronies (1.6% in PSV versus 1.64% in NAVA) despite the proportional assistance applied by NAVA which is known to reduce asynchronies [13, 17, 18, 20, 21, 27, 28].

As previously published by our team [21] and by Piquilloud et al. [13], there was more double triggering in NAVA compared with PSV. The existence of a biphasic aspect of the Eadi signal could explain a successive respiratory cycle during diaphragmatic relaxation (type 1 double triggering) [13], but the occurrence of double triggering was very rare in both modes (0.3% in PSV and 0.4% in NAVA).

Contrary to research on NIV [27] and intubated mechanically ventilated (IMV) patients [10, 13, 18, 29], we did not identify a decrease in the rate of ineffective triggering in NAVA versus PSV. But, as previously noticed, theses asynchronies were rare in our work.

To our knowledge, one study [23] compared patient-ventilator interactions in NAVA versus PSV on tracheotomised patients during the weaning process. The authors assessed the physiological response to varying levels of NAVA and PSV in 13 tracheotomised patients. Patient-ventilator asynchrony was not fully investigated. They only explored ineffective triggering rates, which were not significantly different between the two modes, independently from the level of assistance. This could be explained by the specific interface (tracheostomy) which allows for a decrease in dead space and airway resistance in both ventilator modes. Consequently, it could limit dynamic lung hyperinflation and auto-PEEP.

Our study shows a significant decrease in the total asynchrony index in NAVA versus PSV (2.1% vs 14%, *p* < 0.0001). Thille et al. showed that patients whose asynchrony index was greater than 10% had a longer duration of mechanical ventilation [10] and higher mortality [28]. But the difference on AI only focuses on micro-asynchronies. The micro-asynchrony index was statistically significant lower in NAVA than in PSV (0.35% versus 9.8%).

Despite particular attention being paid to ventilator settings to limit dynamic lung hyperinflation in PSV (personalization of inspiratory trigger, low pressure support level), there was less late cycling in NAVA. In NAVA, the level of assistance is proportional to the patient request and the expiratory trigger is neural, preset to 80% of the Eadi peak. These specificities allow for an optimization of the respiratory parameters from one cycle to the next, with a continuous variability of the pressure support level (cmH_2_O/μV) and the expiratory timing. This result strengthens previous studies that underline the importance of a personal and adapted cycling-off to avoid dynamic hyperinflation, especially in COPD patients [14, 29].

There was little premature cycling in NAVA. This makes sense because, in NAVA, expiratory triggers are systematically neural.

We defined an asynchrony as a delay of 150 ms between the origin of the Eadi signal and the start of the ventilator insufflation, which represents the conscious perception threshold that could be source of discomfort. Consequently, we added a 50-ms safety margin. In other studies, inspiratory delays were measured and the durations were compared based on the ventilator mode [13, 18, 20, 27, 30].

The median respiratory rate (26 versus 28 breaths/min) and the median tidal volume were lower in NAVA than in PSV (5.8 ml/kg versus 6.2 ml/kg) (*p* < 0.001). Furthermore, the Eadi peak was similar in both modes. It has been shown that over-assistance is reflected by a decrease in the Eadi peak [19, 23, 30]. This eliminates the possibility of a disparity in the level of assistance between the two modes. PEEP was lower in NAVA (5 versus 6 cmH_2_O). Better expiratory cycling may limit overdistension and allow for a decrease in PEEP.

Our study has some limitations. This was a single-centre observational study, and was non-randomized. This study demonstrated a reduction in asynchronies in NAVA versus PSV but these results could not lead to an assumption of a benefit concerning weaning duration, hospital length of stay, or survival, mainly due to the small population studied. Prospective clinical studies comparing the weaning from ventilator support in tracheotomised patients with NAVA versus PSV are necessary.

## Conclusion

This is the first study to compare patient-ventilator asynchronies in NAVA and PSV in tracheotomised patients presenting with difficult weaning criteria. There were fewer asynchronies in NAVA with reduced auto-triggering, premature cycling-off, late cycling-off, and inspiratory trigger delay compared with PSV. An asynchrony index greater than 10%, which is related to an increased duration of ventilation, was lower in NAVA. Further studies are required to determine the clinical impact of NAVA on major outcomes in tracheotomised patients.

## Abbreviations

AI: Asynchrony index
COPD: Chronic obstructive pulmonary disease
Eadi: Electrical activity of the diaphragm
ICU: Intensive care unit
NAVA: Neurally adjusted ventilatory assist
PDW: Predicted body weight
PEEP: Positive end-expiratory pressure
PSV: Pressure support ventilation
SAPS: Simplified Acute Physiology Score
VAP: Ventilator-associated pneumonia
VT: Tidal volume
